# Increasing Dengue Incidence in Singapore over the Past 40 Years: Population Growth, Climate and Mobility

**DOI:** 10.1371/journal.pone.0136286

**Published:** 2015-08-31

**Authors:** Claudio Jose Struchiner, Joacim Rocklöv, Annelies Wilder-Smith, Eduardo Massad

**Affiliations:** 1 Oswaldo Cruz Foundation, Rio de Janeiro, Brazil; 2 Institute of Social Medicine, State University of Rio de Janeiro, Rio de Janeiro, Brazil; 3 Epidemiology and Global Health Unit, Department of Public Health and Clinical Medicine, Umeå University, Umea, Sweden; 4 Lee Kong Chian School of Medicine, Nanyang Technological University, Singapore, Singapore; 5 School of Medicine, The University of Sao Paulo and LIM01 HCFMUSP, Sao Paulo, Brazil; 6 London School of Hygiene and Tropical Medicine, London, United Kingdom; Georgia State University, UNITED STATES

## Abstract

In Singapore, the frequency and magnitude of dengue epidemics have increased significantly over the past 40 years. It is important to understand the main drivers for the rapid increase in dengue incidence. We studied the relative contributions of putative drivers for the rise of dengue in Singapore: population growth, climate parameters and international air passenger arrivals from dengue endemic countries, for the time period of 1974 until 2011. We used multivariable Poisson regression models with the following predictors: Annual Population Size; *Aedes* Premises Index; Mean Annual Temperature; Minimum and Maximum Temperature Recorded in each year; Annual Precipitation and Annual Number of Air Passengers arriving from dengue-endemic South-East Asia to Singapore. The relative risk (RR) of the increase in dengue incidence due to population growth over the study period was 42.7, while the climate variables (mean and minimum temperature) together explained an RR of 7.1 (RR defined as risk at the end of the time period relative to the beginning and goodness of fit associated with the model leading to these estimates assessed by pseudo-R2 equal to 0.83). Estimating the extent of the contribution of these individual factors on the increasing dengue incidence, we found that population growth contributed to 86% while the residual 14% was explained by increase in temperature. We found no correlation with incoming air passenger arrivals into Singapore from dengue endemic countries. Our findings have significant implications for predicting future trends of the dengue epidemics given the rapid urbanization with population growth in many dengue endemic countries. It is time for policy-makers and the scientific community alike to pay more attention to the negative impact of urbanization and urban climate on diseases such as dengue.

## Introduction

Dengue is the most important arboviral disease with over half of the world's population living in areas of risk, and about 390 million infections estimated annually[1]. The frequency and magnitude of epidemic dengue have increased dramatically in the last century as both the viruses and the mosquito vectors have expanded geographically in the tropical regions of the world[2]. Reasons for the currently observed and predicted expansion are multi-factorial[3] with climate change, virus evolution and societal factors such as rapid urbanization, population growth as well as global travel and trade thought to be the main factors[4]. Much research has been done to study climate variability [5,6] and its association with dengue epidemics [7–9]. Vectorial capacity of *Aedes* mosquitoes is clearly dependent on temperature [10]. On the other hand, many others have questioned the role of climatic variability as the main driver for the observed expansion of dengue [11–13]. Gubler, for example, suggests that the three principal drivers are urbanization with population growth, globalization and presence of mosquitoes due to the lack of effective control [2]. *Aedes aegypti* is a highly domesticated urban mosquito that prefers to live alongside humans, preferentially feeds on humans and lays eggs in artificial containers made by humans, hence urbanization and population growth provide ideal opportunities for breeding and spread. Globalization on the other hand provides the ideal mechanism for transportation of viruses to regions where dengue is not yet endemic, and can also introduce new serotypes or genotypes into areas that are already endemic, thus triggering new outbreaks[4].

Singapore is an island-city-state in South East Asia that has been dengue endemic for half a century with a well-documented increase in the magnitude of epidemics over the past two decades despite strong political will and sufficient resources for stringent vector control measures [14–17]. The rapid urbanization in Singapore has resulted in doubling of the population over the past two decades. Monthly mean temperature has increased by at least 0.5 Degrees[18]. Singapore has also seen a major increase in international arrivals including arrivals from dengue endemic countries. Serving about 100 international airlines flying to and from some 250 cities in 60 countries and territories worldwide, Changi Airport handles about 6,500 flights every week and over 50 million passengers a year (http://www.changiairport.com/our-business/about-changi-airport/facts-statistics).

Singapore has a good surveillance system with dengue being a legally notifiable disease, with worldwide probably the most reliable data on dengue incidence over decades. Furthermore, the dengue control program in Singapore combines all WHO-recommended control activities, including public health education and community participation, active breeding site detection, environmental management, reactive insecticide fogging, and geo-referenced entomologic and clinical surveillance systems[19]. Hence, Singapore is an ideal place to untangle the following potential individual drivers of dengue resurgence and increasing epidemic peaks: population growth, climate variability and trends, and international air passenger arrivals from dengue endemic countries. The objective of this work was to quantify the relative contribution of three putative drivers to the increasing dengue trends seen in Singapore from 1974 to 2011: population growth, climate variables and numbers of air passengers from dengue endemic countries within the region arriving in Singapore.

## Methods

### Data collection

The incidence of dengue from 1974–2011 was obtained from the Annual Reports published by the Quarantine and Epidemiology Department from 1974–2002 and Ministry of Health from 2003 onwards. More recent data can be viewed at http://www.moh.gov.sg/content/moh_web/home/Publications.html.

Annual Singapore population data were obtained from the Department of Singapore Statistics (http://www.singstat.gov.sg).

We obtained climate data (CRU ts 3.1) from the Climate Research Unit at the University of East Anglia[20]. We extracted climatological data for Singapore longitude 103.75 and latitude 1.25 and aggregated the monthly time series to annual maximum, minimum and mean temperatures and annual cumulative rainfall over the period of 1974 to 2011.

Annual air passenger arrivals to Singapore were obtained from the Singapore tourism board. The predictor variables are available in S1 File and include: Annual Population Size; *Aedes* Premises Index; Mean Annual Temperature; Minimum Temperature Recorded in each year; Maximum Temperature Recorded in each year; Annual Precipitation and Annual Number of Air Passengers/Visitors from South-East Asia where dengue is endemic.

### Multivariate analysis

Associations between dengue incidence and the predictor variables were assessed by multivariate Poisson regression models[21]. The use of generalized linear models (GLM) for modeling binary, categorical and count time series is well established in the statistical literature [22]and inferences based on large sample theory for GLM time series models can be made using standard software for fitting regular GLM models. We fitted our models with function glm available in R language [23]with the extensions described by Harrell[24].

We defined the annual incidence rate (λ), our primary response variable, as the ratio of “total reported dengue cases” by “total population” recorded each year.

$$\lambda \left(t\right)\;=\;\frac{total\;reported\;dengue\;cases\;at\;year\;t}{total\;population\;at\;year\;t}$$ (1)

In order to account for the inter-epidemic behavior in λ(*t*), potentially driven by changes in herd immunity and virology, we used an extended version of the Poisson regression model including auto-regressive terms and harmonic functions to explain and capture cyclic inter-epidemic patterns. Thus, in addition to the predictor variables our model included autoregressive terms of order 3 and terms to account for inter-annual fluctuations with 5 years cycles. The autoregressive structure and period of the model was suggested by previous publications[25] and inspection of the sample autocorrelations of the series and scatterplot matrices relating the variable of interest and their lagged values.

Climate variables are known to be collinear. To address this problem, we compare models in which climate variables are used as input keeping their original formulation or as orthogonal linear combinations obtained by conducting a principal components analysis. In our principal components regression[21], collinear variables *Aedes* Premises Index, Mean Annual Temperature, Minimum Temperature Recorded in each year, and Maximum Temperature Recorded in each year are replaced by the derived linear combinations (scores) given by the first two principal components. We fitted different models including predictor variables (or their scores on the first two principal components), autoregressive terms, and terms to account for inter-annual fluctuations with the following general structure:

$$\begin{array}{l} Y_{t\;}|\;F_{t-1}^{Y,\;\lambda }\;\sim \;Poisson\;\left(\lambda_{t}\right)\\ \text{log}\left(\lambda_{t}\right)=\beta_{0}+\sum_{i=1}^{n}\beta_{i}x_{it}+\sum_{j=1}^{k}\theta_{j}\lambda_{t-j}+\varphi_{1}\text{sin}\left(2\pi \omega t+\phi \right)+\varphi_{2}\text{cos}\left(2\pi \omega t+\phi \right) \end{array}$$ (2)

where *Y*_*t*_ denotes the time series process such that the observed number of dengue cases is a realization of this random process, $F_{t-1}^{X,\lambda }$ contains the information about the past of the process up to time *t*-1, *β*_0_ is the intercept, *β*_*i*_ are the regression coefficients for each variable X_i_ (annual population; mean, maximum and minimum temperature; annual rainfall; mean annual *Aedes* premise index; annual air passenger arrivals from South East Asia) or the appropriate PCA score, *θ*_*j*_ are the autoregressive components of the lag of incidence λ_t-j_, and *φ*_*s*_, *s* = 1,2 are the coefficients that weight the role of inter-year cycles. Parameters *ω* and *φ* allow for cycles with a 5-year period. We derived the best fitting model according to the Akaike Information Criterion (AIC), the Pseudo-R^2^[26], and assessed the goodness of fit of the predictions graphically. We used log(population) as an offset in the regression models to adjust for the population at risk by time t.

In the subsequent analysis, we studied the trends in the predictor variables over the study period and attributed the associated change according to the significant trend estimates from the start of the study period to the end of the study period. This step was taken in order to estimate the different contributions of the predictor variables to the dengue trends and epidemics in Singapore over the last 4 decades. In these analyses we established regression models using the predictor variables identified in model (2) as response variables in a linear regression model including time as predictor variable and a Gaussian distribution error:

$$\begin{array}{lll} X & \;=\; & Normal\;\left(\mu ,\sigma \right)\\ x_{i} & \approx & \gamma_{i}time_{t}+error_{t} \end{array}$$ (3)

where *x*_*i*_ are the predictor variable from previous models, time is an annual time variable, and γ_i_ are the coefficients for the annual trend in the predictor variable.

In a Poisson regression setting, the effect of a covariate of interest *i* is usually given as the rate ratio per unit change (RR_i_) of the covariate and expressed as RR_i_ = exp(β_i_). Measures of effect expressed as rate ratios per unit change are non-intuitive to compare directly when the covariates span different ranges of value (e.g. the variability of the variables could be magnitudes different). For example, a unit change in population size is one individual and a unit change in temperature is one degree. Therefore, the impact on the disease rate caused by adding one individual to the population cannot be compared to the impact of increasing one degree in temperature. We have then re-expressed our measures of effect as follows in order to take this behavior into account by stable estimates of the amount of change occurring in the study period in the predictor variables of model (2) through establishing model (3).

We estimated the incremental changes according to the trend estimates over the study period to the different statistically significantly predictor variables in model (2) using the beta estimates (RRs) from model (2), in combination with the change in the predictor variable over the study period estimated through Eq (3) as:

$$\gamma_{i}*\left(t_{end}-\;t_{start}\right)$$ (4)

This computation provide robust estimates of the change in the predictor variable from the start of the period to the end of the period while removing random inter-annual variability. The relative risk related to variable *i* from model (2) associated with the trend change in variable *i w*as estimated through combining information from models (2–4) to compute the rate ratio of the predictor variable on the dengue incidence over the study period through:

$$RR_{i}=\;(exp\left(\beta_{i}*\gamma_{i}*\left(t_{end}-\;t_{start}\right)\right))$$ (5)

The RR_i_ thus estimates the relative risk according to the increasing trends in the predictor variable over the study period, describing how many times larger the risk is from the beginning to the end of the study period given the change in trend in one predictor variable. A combined measure of the drivers rate ratios can be derived by multiplication different drivers. Their degree of contribution to the increasing dengue incidence in Singapore can be estimated using a relative contribution framework according to:

$$\text{Proportion explained by }RR_{i}\;=\;RR_{i}/\Sigma RR_{i}$$ (6)

*over all i´s*.

The calculations were performed in R language [23].

## Results

**Fig 1:**
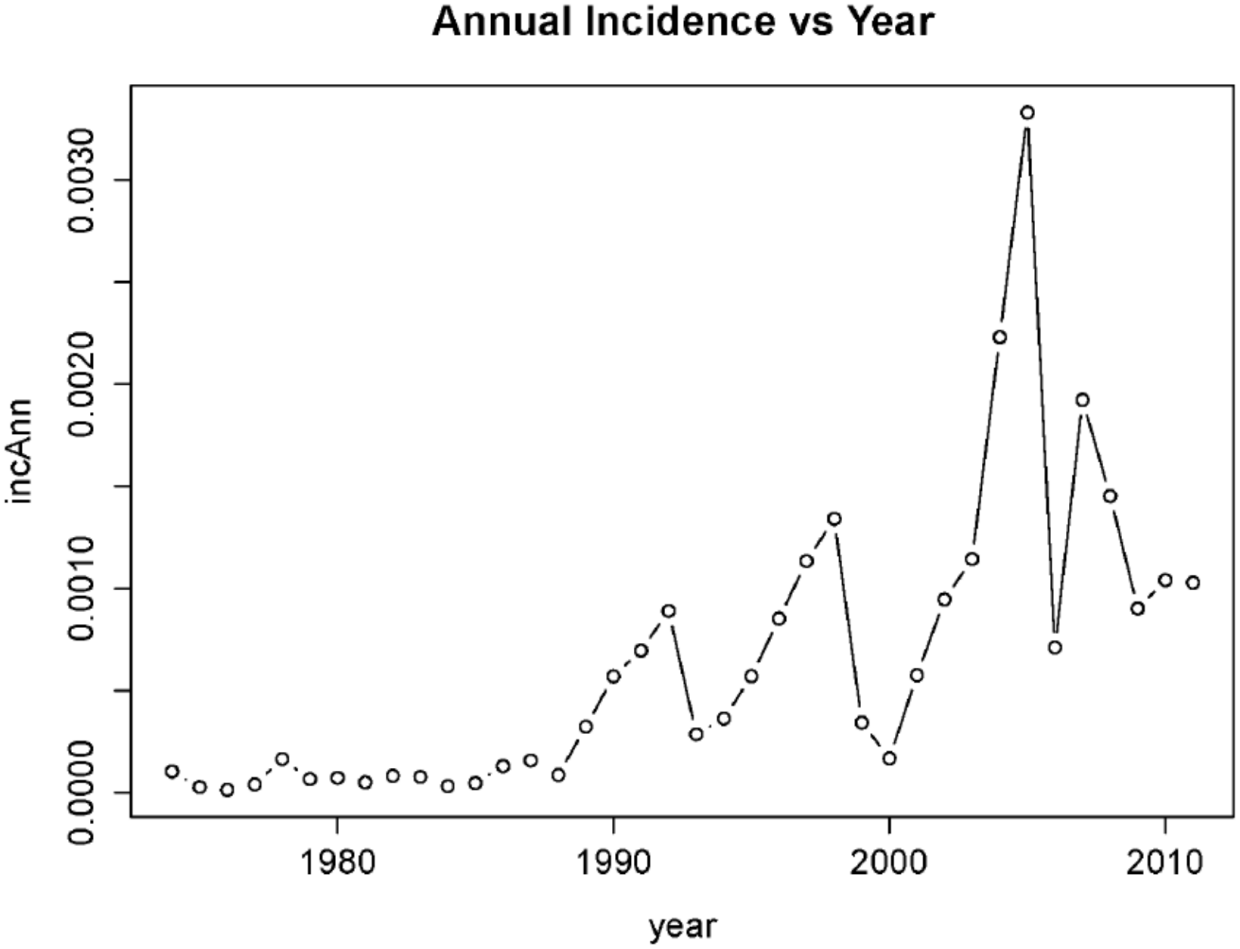
Time series of annual incidence of dengue in Singapore, 1974–2011.

The annual dengue incidence increased dramatically over the study period (Fig 1). The incidence λ(*t*) shows an inter-annual cyclic component. Table 1 compares the various models derived from the general structure described by expression 2. The objective in comparing these models is twofold. First we seek to perform regular model diagnosis routines (including the stability of regression parameter estimates and error structure) and second, we seek the most parsimonious model that best fit the data.

**Table 1:** Model comparison.

Model	AIC	MSE	R2	*β*pop	S.E.	Wald Z	p-values
M1	16993.4	1706615	0.83	1.48e-06	4.68e-08	31.62	<0.0001
M2	21834.9	3266926	0.68	1.34e-06	3.75e-08	35.61	<0.0001
M3	17568.4	1879272	0.82	1.73e-06	3.54e-08	48.80	<0.0001
M4	20721.4	2414094	0.76	9.01e-07	3.06e-08	29.46	<0.0001
M5	17018.9	1740497	0.83	1.45e-06	5.50e-08	26.37	<0.0001

Note: M1- model complete with all variables expressed with their original values; M2 –variables maxT, minT, meanT and premise replaced by the scores in PCA1 and PCA2; M3 –same as M1 but dropping the autoregressive component; M4 –same as M1 but dropping the cyclic component; M5 –same as M1 but removing the variables that did not achieve significance (maxT); *β*pop, S.E., Wald Z and p-values refer to the estimates of *β* for variable “Total Population” under the various models.

Model M5 displays the best compromise among the properties compared and has as inputs auto-regressive terms with lags 1–3, *Aedes* premise index, min and mean temperature, precipitation, total population (in addition to offsetting population at risk), and international visitors. The coefficients and confidence intervals with these estimates are shown in Table 2. Mean and minimum temperature, as well as *Aedes* premise and total population had a significant and positive effect on dengue incidence, while rainfall and annual visitor arrivals were not associated with increasing dengue incidence.

**Table 2:** Estimates of Regression coeficients of the best fitting model (M5).

Variable	Coefficient β_i_	Standard Error	Wald Z	p-values
Intercept	-30.88	1.2048	-25.63	<0.0001
Premises index	0.08	0.0157	5.20	<0.0001
Mean Temperature	0.34	0.0283	11.84	<0.0001
Minimum Temperature	0.63	0.02342	6.88	<0.0001
Precipitation	-2.97 x 10^-5^	0.0000014667	-12.21	<0.0001
Visitors from SE Asia	-6.92 x 10^-7^	0.0000000280	-20.26	<0.0001
Population Size	1.24 x 10^−6^	0.0000000561	22.08	<0.0001
log (Incidence—Lag1)	0.17	0.0167	10.49	<0.0001
log (Incidence—Lag2)	-0.03	0.0083	-3.10	0.0019
log (Incidence—Lag3)	0.16	0.027	12.29	<0.0001
sin(*Year*)	0.02	0.0077	2.62	0.0087
cos (*Year*)	0.32	0.027	6.60	<0.0001

**Fig 2:**
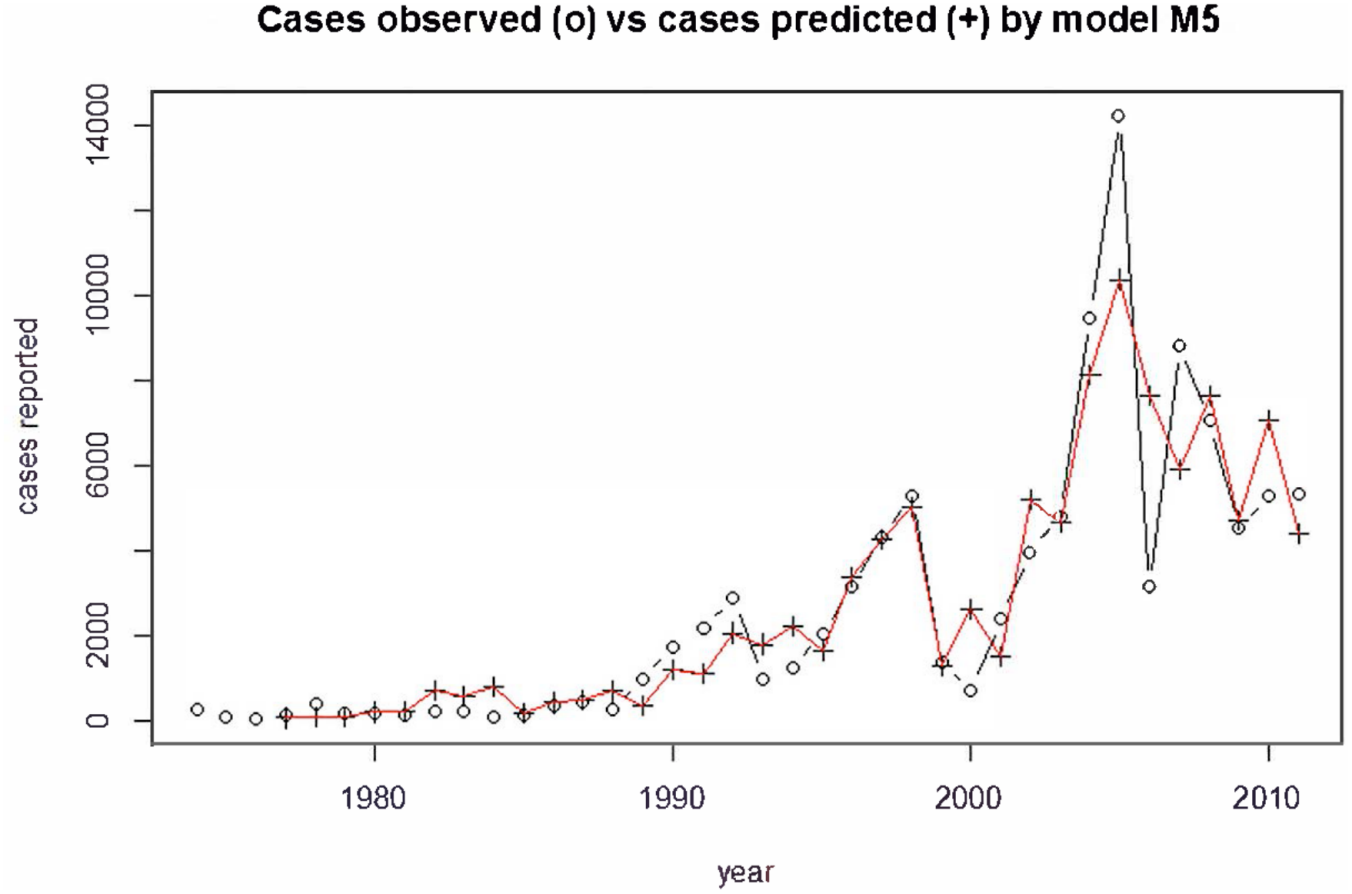
Predicted annual disease cases from the final model (M5) together with the observed/reported number of cases.

The second best fitting model was a model including the same variables plus the maximum temperature. The model fits of these models are presented in Table 1. It shows a comparison of the goodness of fit: AIC, mean square error (MSE) and R-square (R2). The two models describe the data well with an R^2^ greater than 0.8. This can be visually confirmed after inspection of Fig 2 where the observed and predicted annual cases are plotted. The simpler model (M5) was chosen on the basis of a combined judgment of R^2^ and AIC. According to AIC the model fit was better with maximum temperature (although this variable was not statistically significant). However, the R^2^ did not show any difference and therefore the simpler model was chosen as the final model.

### Contribution of risk fractions related to time trends over the study period

By inspecting Tables 3 and 4 with the Poisson model’s coefficients (incidence rate ratios) one can conclude that variables mean and minimum temperatures and population are important in determining the incidence of dengue in the period analyzed. We note here that population is also being offset in the models so its associated effect should be viewed as an indicator of population density.

**Table 3:** Independent Variables of the Poisson Regression Model with the respective coefficients and incidence ratios.

Variable	Coefficient β_i_ per 1 unit of predictor variable	Incidence Ratios e^βi^
Premises index	8.16 x 10^-2^	1.085 per unit increase in the index
Mean Temperature	3.35 x 10^-1^	1.398 per°C
Minimum Temperature	6.29 x 10^-1^	1.876 per°C
Precipitation	-2.97 x 10^-5^	0.997 per 100 mm precipitation
Visitors from SE Asia	-6.92 x 10^-7^	0.933 per 100,000 travellers
Population Size	1.24 x 10^−6^	1.132 per 100,000 populations

**Table 4:** Relative risks and associated risk fractions (%) associated with each driver associated with the trend change in that specific variables over the study period.

Variable	RR (relative contribution, %)
**Population**	42.7 (86)
**Min. Temp.**	3.9
**Mean Temp.**	1.8
**Climate Combined**	7.1 (14)

**Fig 3:**
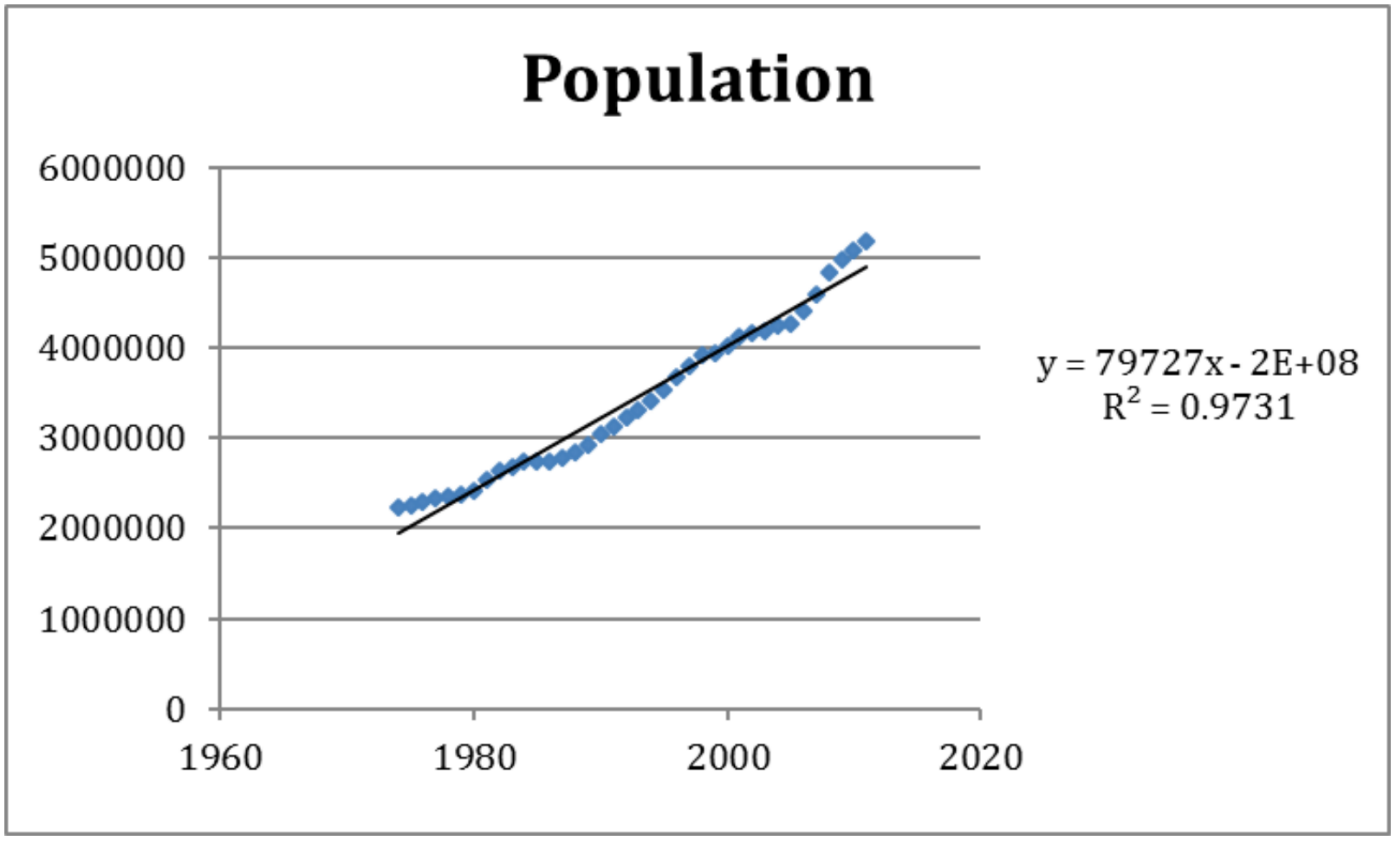
Temporal variation of population size over the period studied.

**Fig 4:**
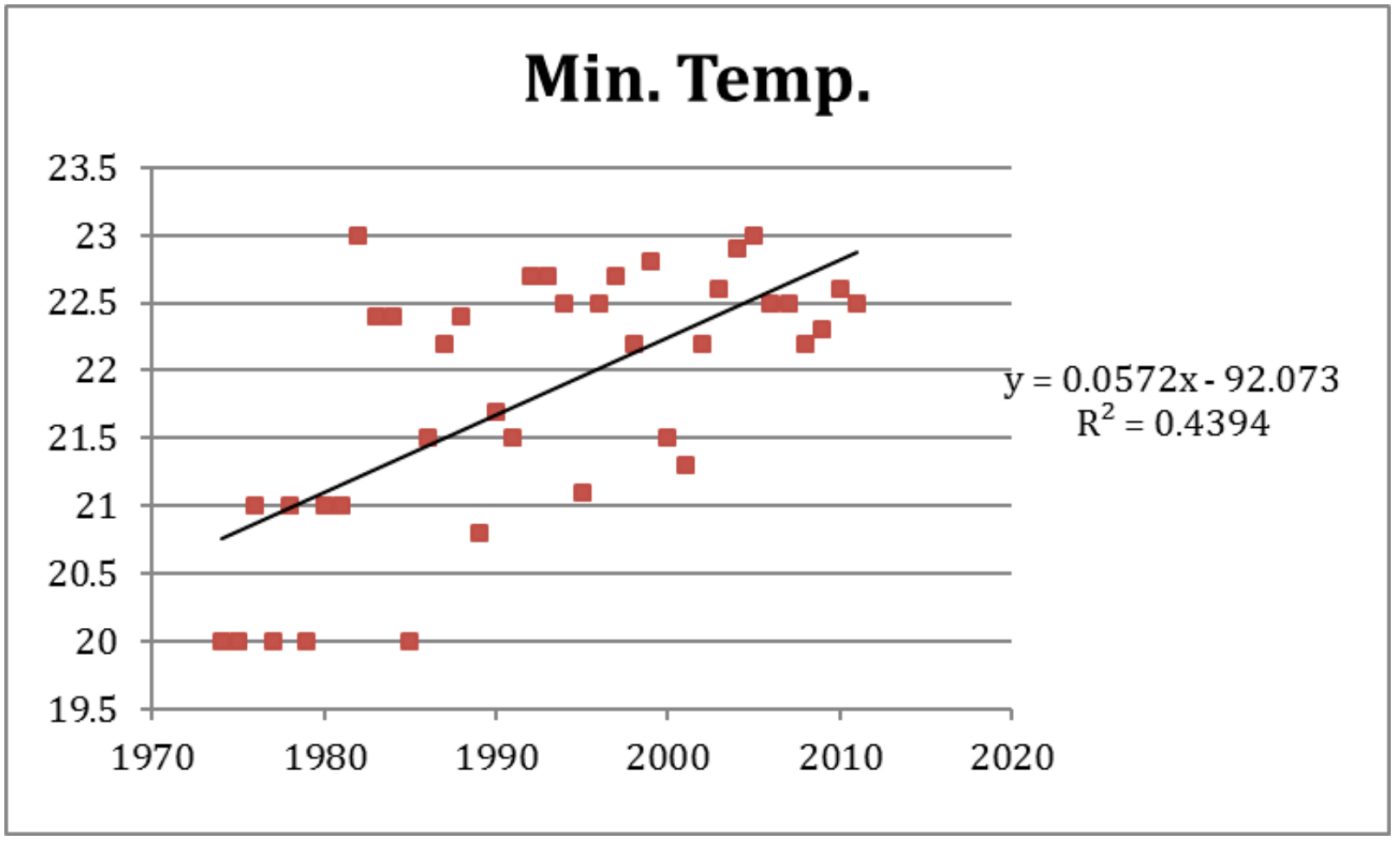
Temporal variation of minimum temperature over the period studied.

**Fig 5:**
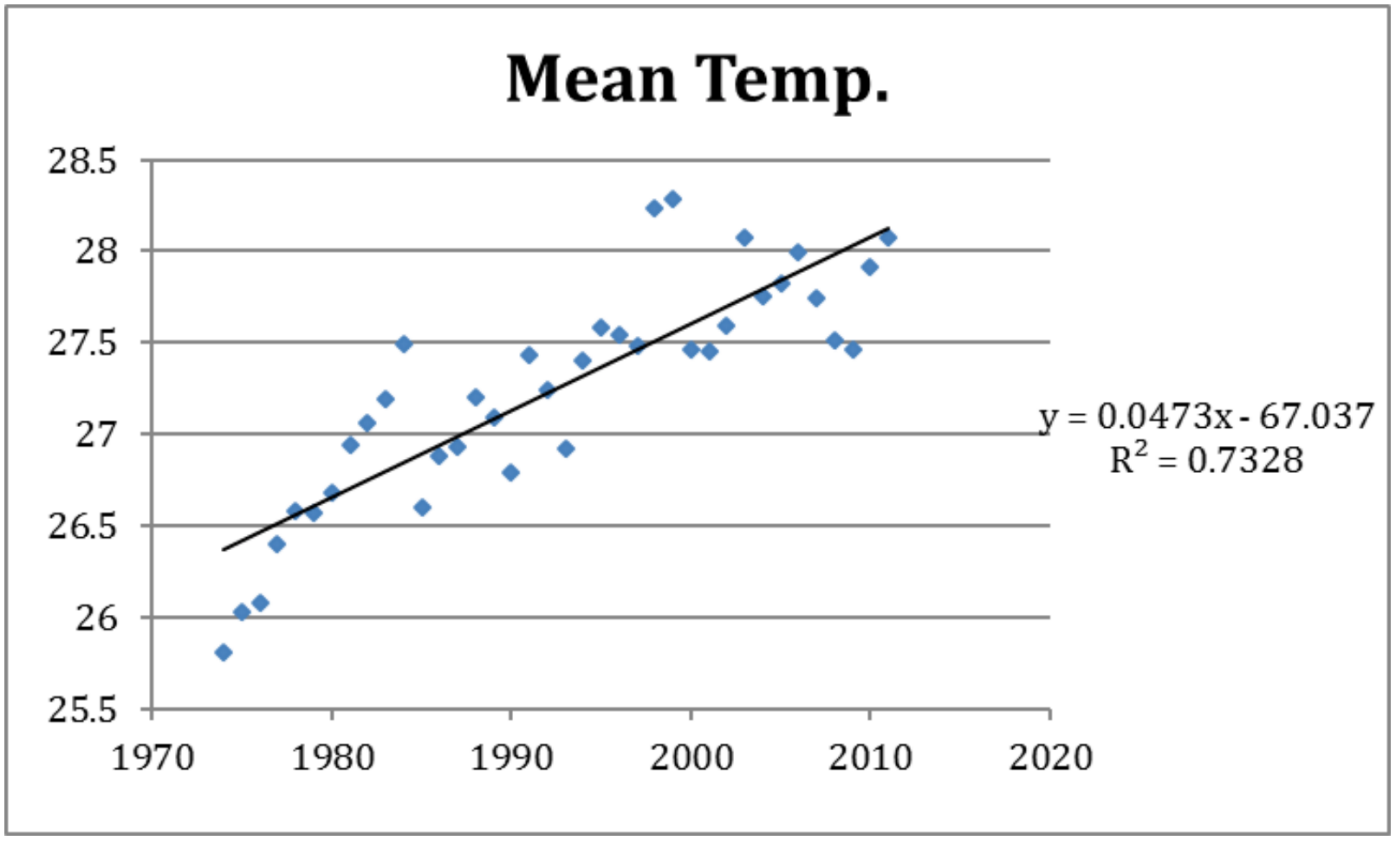
Temporal variation of mean temperature over the period studied.

The increasing trends in the predictor variables of importance were estimated to an: 79,727 persons population increase per year (Fig 3), a 0.057°C increase in minimum temperature per year (Fig 4), and a 0.047°C increase in mean temperature per year (Fig 5). Over the study period of 38 years these coefficients translate to a total growth in population of around 3 million, and a warming of 2.2°C for minimum temperature and 1.8°C for mean temperature according to Eq (3). Adding this increase to the estimated effects of these variables on dengue incidence according to model M5 we estimate the amount of change over the study period in the incidence rate ratio. The relative contribution to the change associated with the change over the study period are presented in Table 4. The relative risk (RR) of the increase in dengue incidence due to population growth over the study period was 42.7, while the climate variables (mean and minimum temperature) together explained a RR of 7.1.

When comparing the importance of the different categories of drivers, the relative proportion of the increasing trends explained by population growth as compared to the population growth combined with climate, the population growth amounts to 86% of the combined change, and the relative proportion explained by climate variables explain the residual 14%. Both drivers thus amount to have increased the disease burden of dengue in Singapore significantly.

## Discussion

Of the three putative drivers for dengue expansion, population growth was by far the leading independent factor associated with the increase in dengue cases observed in Singapore over the past 40 years, followed by mean temperature change. Air passenger arrivals from dengue endemic countries had no effect. Poisson regression analyses with autoregressive terms allowed us to estimate the effect of population growth: for every 100,000 persons population increase per year, dengue cases increased by 13%. The incidence rate ratio related to the total change in population size, of approximately 3 million populations, and the incidence of dengue over the study period amounted to 42.7. We found also mean and minimum annual temperature to have increased the incidence rate of 39.8% and 87.6% per°C increase, and amounting to a combined incidence rate increase of 7.1 for the approximately 2°C temperature change over the study period.

Based on our calculations comparing the drivers to each other in a relative fashion, the increasing trends of dengue infections in Singapore were 86% attributed to population growth and 14% related to the change in temperature.

We took into account the usual measures of goodness of fit as well as diagnostic procedures when comparing each model fitting exercise. Our modeling strategy also considered an extension of the generalized linear models framework to a time series framework. By extending this framework, we were able to express current incidence as a function of past incidence, an approach that is important as it indirectly addresses the role of herd immunity in dengue dynamics. We also explored the presence of potential disease cycles by the use of trigonometric functions. Additional model components, e.g. PCA scores, account for the collinear behavior of climate variables and mosquito density. By considering all these issues, we were able to robustly isolate the effect of total population size as a driver of dengue epidemics. It is important to note that the increase in dengue cases related to population growth was not just a simple matter of "more people, more cases" e.g. a linear function, but our findings show that the incidence of dengue cases increased as an exponential function of population size in the study period.

The notion that population growth is an important driver for dengue is also biologically plausible. Urbanization substantially increased the density, larval development rate, and adult survival time of Aedes mosquitoes in a study in China[27]. Rapid dispersal of oviposition is driven by the availability of oviposition sites that are more readily available in urbanized conditions[28]. Given the close proximity of humans in cities, Aedes mosquitoes can bite several persons within one blood meal, thus amplifying dengue transmission dynamics[29]. Urbanization drives dengue epidemics because dengue viruses have fully adapted to the human-*Aedes aegypti*-human transmission cycle, which can flourish in crowded human populations that live in intimate association with anthropophilic *Aedes* mosquito populations[30].

Our models may also explain the paradoxical findings by Egger et al [19] that dengue incidence in Singapore is increasing despite the fact that Singapore is one of the few settings that have recorded sustained suppression of the vector population over decades[31]. Close proximity of humans in overcrowded urbanized settings may overcome the reduced force of infection under highly effective vector control programs with documented reduction in *Aedes* mosquito household indices.

Dengue infections are climate sensitive, and increasing mean temperatures have been documented to correlate with increased dengue incidence and outbreaks in Singapore [9]. Although in our study the contribution of the temperature trends in Singapore played a lesser role in the dengue transmission trends over decades compared with population growth, our findings show an independent and significant increase in dengue incidence associated with mean temperatures. The temperature increase that has been documented over time in Singapore is likely to be partly due to the heat effect of urbanization and partly due to global climate change. Hence, population growth also has an indirect effect on temperature increase through urbanization, thus the contributing effect of population growth on dengue incidence also explains some of the temperature effect in our modeling estimates.

The overall low contribution (14%) of climate parameters in Singapore when calculating the Population Association Fraction is most likely due to the fact that Singapore overall does not exhibit much intra-annual and inter-annual climate variability due to its all-year round hot and humid climate. The relative contribution of temperature and other climate factors may be higher in other settings that have a more distinct seasonality and inter-annual variation. It is also important to point out that projected increasing temperature levels in the future will not necessarily lead to increased dengue epidemic potential in Singapore as temperature driven vectorial capacity is not linear: temperatures above a certain threshold (e.g. mean temperature far above 30 C) can lead to severely reduced vectorial capacity of Aedes mosquitoes resulting in reduced dengue epidemic potential [32].

We hypothesized that the higher the air passenger volume from dengue endemic countries, the higher the probability of introduction of dengue virus serotypes and novel genotypes. However, our findings show that air passenger volume from dengue endemic countries of SE Asia into dengue endemic Singapore was not correlated with increasing dengue incidence. A plausible explanation could be that the vast majority of travelers from these areas display high pre-existing dengue immunity. Although introduction of novel dengue virus genotypes may result in epidemics [33–38], not every new virus or clade introduction results in dengue epidemics[39]. In other words, sometimes, novel introductions do not result in an outbreak, instead, genotypic changes over time within a country independent of new introduction from the outside can lead to local epidemics. To specifically study the role of dengue virus introduction, employing molecular epidemiological studies with dengue virus sequencing would be a better approach to determine the origin of a newly imported virus.

Our study has several limitations. First of all, we rely on dengue notifications that are subject to reporting bias. Increasing notifications over the past decades due to increasing awareness of dengue could have led to an overestimation of increasing trends in dengue incidence. Second, we only focused on three putative drivers. We did not deem it necessary to include studying vector densities as a predictive factor because of the documented low and even decreasing vector indices in Singapore[31]. We therefore also did not include changing patterns in vector control measures over the past 40 years. However, serotypes and co-circulation of multiple serotypes would have been a parameter that theoretically could be an independent and significant predictive factor for dengue incidence. But we were not able to include co-circulation or new introductions of serotypes due to the lack of such information over our historically long time span of 40 years. Similarly, other variables that were not taken into account in our modeling efforts could bias the estimates from this study. However, the relatively high explanatory degree of the models, and the plausibility of the mechanisms related to the variables studied support the validity and importance of our findings. Third, collinear variables represent an important source of instability in parameter estimation when they enter simultaneously in the regression model. Climate variables are well known for being potentially collinear. Therefore, we entertained models that have as input climate variables in their original formulation or, alternatively, as orthogonal scores obtained from a principal components analysis.

In conclusion, this study may serve as one of the best proxies we have today to understand the relative contribution and impact of three potential drivers responsible for dengue incidence in endemic settings: population growth, climate change and globalization. Societal effects such as population density, population growth and urbanization appear to be the leading drivers for the observed rise in dengue cases in Singapore over the past 40 years, much more than meteorological parameters such as temperature and rainfall, and climate change at large. Population growth is also more important than global mobility in terms of incoming air passenger arrivals, at least in an endemic setting such as Singapore. Our findings have significant implications for predicting further trends of the dengue given the rapid urbanization patterns worldwide. The population size in South East Asia, which carries the main burden of dengue, with currently an estimated population at around 580 million, has increased by more than 30% since 1990[40]. Unprecedented urbanization in South East Asia began in the years following World War II and coincided with a remarkable economic boom[41]. Cities like Bangkok, Manila, and Jakarta exploded in population growth, most of it unplanned[41]. It was at the same time that epidemic DHF emerged, first recognized in Manila in 1953–1954, followed by Bangkok in 1958[30]. The American region became highly urbanized in the 1970s and today, over 75% of the population live in urban areas, nearly all of which have been re-infested with *Aedes aegypti* [42]. It is time for policy-makers and the scientific community alike to pay more attention to the negative impact of urbanization on diseases such as dengue.

## Supporting Information

S1 File Variables analysed relative to dengue outbreaks in Singapore from 1974 to 2011. (DOCX)
