# Supplementary material for: Increasing Dengue Incidence in Singapore over the Past 40 Years: Population Growth, Climate and Mobility
Source: PLoS One. 2015 Aug 31;10(8):e0136286. doi: 10.1371/journal.pone.0136286 (PMC4554991; doi:10.1371/journal.pone.0136286)
Supplement: S1 File — (DOCX) [file pone.0136286.s001.docx]

Supplement 1

| **S1 File. Variables analysed relative to dengue outbreaks in Singapore from 1974 to 2011.** | | | | | | | | | |
| --- | --- | --- | --- | --- | --- | --- | --- | --- | --- |
| **Year** | **Total Population** | **Total reported dengue cases** | **incidence** | **Aedes premise index** | **Meantemp** | **Min temp** | **Max temp** | **Precipit** | **Visitorsfrom SE Asia** |
| 1974 | 2229800 | 230 | 0.00010315 | 5 | 25.8038 | 20 | 33 | 0 | - |
| 1975 | 2262600 | 59 | 2.6076E-05 | 6 | 26.0277 | 20 | 36 | 0 | - |
| 1976 | 2293300 | 30 | 1.3082E-05 | 3 | 26.0758 | 21 | 33 | 0 | - |
| 1977 | 2325300 | 93 | 3.9995E-05 | 4 | 26.4027 | 20 | 37 | 0 | - |
| 1978 | 2353600 | 387 | 0.00016443 | 5 | 26.5806 | 21 | 33 | 0 | - |
| 1979 | 2383500 | 157 | 6.587E-05 | 4 | 26.5682 | 20 | 33 | 0 | - |
| 1980 | 2413900 | 173 | 7.1668E-05 | 4 | 26.6847 | 21 | 33 | 0 | - |
| 1981 | 2532800 | 126 | 4.9747E-05 | 4 | 26.9434 | 21 | 35 | 0 | - |
| 1982 | 2646500 | 216 | 8.1617E-05 | 4.5 | 27.0656 | 23 | 33 | 0 | - |
| 1983 | 2681100 | 205 | 7.6461E-05 | 4 | 27.1898 | 22.4 | 33.4 | 0 | - |
| 1984 | 2732200 | 86 | 3.1476E-05 | 4 | 27.4909 | 22.4 | 34.8 | 0 | - |
| 1985 | 2736000 | 126 | 4.6053E-05 | 3.5 | 26.6058 | 20 | 33 | 0 | - |
| 1986 | 2733400 | 354 | 0.00012951 | 2.5 | 26.8817 | 21.5 | 33 | 1511.5 | - |
| 1987 | 2774800 | 436 | 0.00015713 | 2.5 | 26.9351 | 22.2 | 33.4 | 3600.9 | - |
| 1988 | 2846100 | 245 | 8.6083E-05 | 2.5 | 27.2004 | 22.4 | 33 | 3007.9 | - |
| 1989 | 2930900 | 944 | 0.00032209 | 1 | 27.0959 | 20.8 | 33.2 | 3770.2 | - |
| 1990 | 3047100 | 1733 | 0.00056874 | 0.8 | 26.7909 | 21.7 | 33.2 | 2268.7 | 1706648 |
| 1991 | 3135800 | 2179 | 0.00069488 | 0.59 | 27.4361 | 21.5 | 33.8 | 844 | 1842709 |
| 1992 | 3232100 | 2878 | 0.00089044 | 0.96 | 27.2415 | 22.7 | 33 | 1239.5 | 1975755 |
| 1993 | 3315400 | 946 | 0.00028534 | 1.18 | 26.9197 | 22.7 | 33.7 | 5887.4 | 2184693 |
| 1994 | 3421100 | 1239 | 0.00036216 | 1.06 | 27.4082 | 22.5 | 33.1 | 9831.3 | 2227532 |
| 1995 | 3525600 | 2008 | 0.00056955 | 1.16 | 27.5875 | 21.1 | 33.2 | 8085.3 | 2288674 |
| 1996 | 3670400 | 3128 | 0.00085222 | 1.54 | 27.5472 | 22.5 | 33.8 | 9755.4 | 2351160 |
| 1997 | 3793700 | 4300 | 0.00113346 | 1.03 | 27.4866 | 22.7 | 33.6 | 12160.1 | 1887616 |
| 1998 | 3922000 | 5258 | 0.00134064 | 1.03 | 28.234 | 22.2 | 34.4 | 6229.5 | 2223971 |
| 1999 | 3950900 | 1355 | 0.00034296 | 0.59 | 28.2907 | 22.8 | 35.1 | 76146.9 | 2427668 |
| 2000 | 4017700 | 673 | 0.00016751 | 1.3 | 27.4623 | 21.5 | 33.8 | 19185.8 | 2522922 |
| 2001 | 4131200 | 2372 | 0.00057417 | 1.54 | 27.4541 | 21.3 | 33.6 | 30394.4 | 2532887 |
| 2002 | 4171300 | 3945 | 0.00094575 | 1.97 | 27.5972 | 22.2 | 33.5 | 6515.1 | 2307229 |
| 2003 | 4185200 | 4788 | 0.00114403 | 2.02 | 28.0722 | 22.6 | 34.1 | 3958.3 | 3085876 |
| 2004 | 4240300 | 9459 | 0.00223074 | 2.69 | 27.7575 | 22.9 | 33.9 | 5376.1 | 3341721 |
| 2005 | 4265800 | 14210 | 0.00333115 | 1.15 | 27.821 | 23 | 34.3 | 5940.5 | 3577365 |
| 2006 | 4401400 | 3126 | 0.00071023 | 0.18 | 27.9935 | 22.5 | 34.8 | 4248.8 | 3724736 |
| 2007 | 4588600 | 8826 | 0.00192346 | 0.68 | 27.747 | 22.5 | 34.2 | 6327.6 | 3571408 |
| 2008 | 4839400 | 7032 | 0.00145307 | 0.66 | 27.5156 | 22.2 | 33.5 | 6757.4 | 3684848 |
| 2009 | 4987600 | 4498 | 0.00090184 | 0.34 | 27.4593 | 22.3 | 33.6 | 5306.9 | 4819751 |
| 2010 | 5076700 | 5279 | 0.00103985 | 0.35 | 27.9114 | 22.6 | 34 | 4289.9 | 5414250 |
| 2011 | 5183700 | 5330 | 0.00102822 | 0.35 | 28.0715 | 22.5 | 35.1 | 4514.1 | 6008749 |
